# Left Atrial Appendage Closure: Past, Present, and Future

**DOI:** 10.19102/icrm.2026.17095

**Published:** 2026-09-15

**Authors:** Elio Zito, Pamela Horton Embrey, Vincenzo Mirco La Fazia, Carola Gianni, Sanghamitra Mohanty, Claudio Tondo, Andrea Natale, Rodney Horton

**Affiliations:** 1 University of Milan, Milan, Italy; 2 Texas Cardiac Arrhythmia Institute, St. Davids Medical Center, Austin, TX, USA; 3 Division of Internal Medicine, The University of Texas Health Science Center at Houston, Houston, TX, USA; 4 Università Tor Vergata, Rome, Italy; 5 Centro Cardiologico Monzino, IRCCS, Milan, Italy; 6 Interventional Electrophysiology, Scripps Clinic, San Diego, CA, USA; 7 Metro Health Medical Center, Case Western Reserve University School of Medicine, Cleveland, OH, USA

**Keywords:** Atrial fibrillation, left atrial appendage closure, WATCHMAN, AMULET

## Abstract

Left atrial appendage closure (LAAC) has emerged as an important alternative strategy for stroke prevention in patients with non-valvular atrial fibrillation (NVAF), particularly when long-term oral anticoagulation (OAC) is contraindicated or poorly tolerated. Initially developed as a surgical technique based on ligation or excision of the left atrial appendage, LAAC has progressively shifted toward refined, minimally invasive transcatheter approaches. Randomized and observational data have established non-inferiority to warfarin, and non-inferiority compared with direct oral anticoagulants has also been suggested in selected high-risk cohorts and is currently under prospective evaluation in pivotal trials. At the same time, indications for LAAC are expanding beyond the classical high-bleeding-risk population, now encompassing patients undergoing atrial fibrillation (AF) ablation and those with only moderate thromboembolic risk but intolerance to OAC. Parallel advances in device design aimed at improving anatomical conformability, sealing performance, and surface biocompatibility seek to reduce peri-device leak (PDL), device-related thrombus (DRT), and bleeding and may allow for simplified or shortened post-procedural antithrombotic regimens. This narrative review summarizes the historical evolution of LAAC from surgical to contemporary transcatheter solutions, outlines key clinical evidence versus medical therapy, and discusses emerging implant-based and implant-free technologies. As evidence accumulates from ongoing randomized controlled trials, LAAC may transition from a niche intervention to a more widely adopted, guideline-supported component of AF management. However, broader implementation will depend on robust demonstration of long-term safety and efficacy, including clarification of PDL-, DRT-, and bleeding-related trade-offs, to define when LAAC can serve not only as an alternative to OAC but also as a tailored option for selected NVAF patients.

## Introduction

Atrial fibrillation (AF), the most common cardiac arrhythmia worldwide, is associated with a fivefold increased risk of ischemic stroke.^1^ In patients with non-valvular AF (NVAF), the left atrial appendage (LAA) is the primary site of thrombus formation, accounting for >90% of cardiac emboli.^2^ Traditionally, long-term oral anticoagulation (OAC) has represented the cornerstone of stroke prevention therapy in this population.^3,4^ However, a significant proportion of patients either have contraindications to anticoagulation, such as previous major bleeding or high hemorrhagic risk, or experience complications that limit its long-term use. In response to these limitations, mechanical exclusion of the LAA has emerged as an attractive alternate strategy to reduce the risk of stroke.^3–5^ Over the decades, techniques have evolved from empirical surgical ligation to percutaneous, catheter-based LAA closure (LAAC), allowing for minimally invasive occlusion in patients who may not otherwise tolerate surgery or OAC. This paradigm shift has been supported by a growing body of evidence from randomized controlled trials (RCTs), registry data, and long-term observational studies, many of which have demonstrated non-inferiority to warfarin and favorable safety profiles **(Tables 1 and 2)**. Today, the landscape of LAAC is rapidly expanding. This new generation of devices offers improved conformability, enhanced sealing capabilities, and reduced thromboembolic and hemorrhagic complications. Clinical trials are also starting to explore the role of concomitant LAAC in patients undergoing catheter ablation and in those without absolute contraindications to anticoagulation. This narrative review provides a comprehensive overview of the evolution of LAAC, including current evidence, emerging technologies, and ongoing trials that may redefine its future role in preventing cardioembolic stroke in AF management.

**Table 1:** Timeline of Completed and Ongoing RCTs and Observational Registry Evidence for Surgical and Percutaneous LAAC Published over the Last 20 Years

Year	Study Name	Endpoint	Device
2003	LAAOS I	Investigated surgical LAA closure feasibility during CABG	—
2009	PLAATO study	Evaluated percutaneous LAA occlusion feasibility in AF patients unsuitable for OAC	PLAATO device (discontinued)
2009	PROTECT AF	Compared LAAC to warfarin in NVAF	WATCHMAN™ 2.5
2013	LAAOS II	Surgical closure safety in cardiac surgery patients	—
2014	PREVAIL	Validated WATCHMAN™ vs. warfarin with procedural refinements	WATCHMAN™ 2.5
2015	EWOLUTION Registry	Registry-based evaluation of WATCHMAN™ in routine care	WATCHMAN™ 2.5
2018	Global Amulet Registry	Evaluated procedural success with Amulet™ in practice	Amulet™
2019	CAP and CAP2	Long-term analysis after PROTECT AF and PREVAIL trials	WATCHMAN™ 2.5
2020	PRAGUE-17	Compared LAAC vs. DOACs in high-risk NVAF	WATCHMAN™ 2.5/FLX™, Amulet™
2020	ASAP Study	LAAC outcomes in OAC-ineligible patients	WATCHMAN™ 2.5
2020	NCDR LAAO Registry	Assessed real-world procedural success and safety	WATCHMAN™ 2.5
2021	LAAOS III	Evaluated stroke prevention with surgical LAA closure	—
2021	Amulet IDE	Compared Amulet™ vs. WATCHMAN™	Amulet™ vs. WATCHMAN™ 2.5
2021	PINNACLE FLX	Assessed safety of next-generation WATCHMAN™	WATCHMAN FLX™
2022	SWISS-APERO	Compared Amulet™ vs. WATCHMAN FLX™ anatomically	Amulet™ vs. WATCHMAN FLX™ (>80%)/WATCHMAN™ 2.5
2022	PROTECT/PREVAIL 5Y	Long-term follow-up of earlier RCTs	WATCHMAN™ 2.5
2022	SURPASS Registry (1Y)	Evaluated real-world outcomes of >17,000 WATCHMAN FLX™ implants	WATCHMAN FLX™
2023	OPTION Trial	Compared LAAC vs. OAC after AF ablation	WATCHMAN FLX™
2025	SWISS-APERO 3Y	Extended follow-up of anatomical and ischemic outcomes	WATCHMAN FLX™ vs. Amulet™
2025	CLOSURE-AF	Compared LAAC vs. optimal medical therapy in high-bleeding-risk patients	WATCHMAN FLX™, Amulet™
Est. 2026–2027	COMPARE LAAO	Comparing LAAC vs. SAPT/DAPT in patients with contraindications to OAC	WATCHMAN FLX™, Amulet™
Est. 2027–2028	CHAMPION-AF	Comparing LAAC to DOACs	WATCHMAN FLX™
Est. 2027–2028	CATALYST	Comparing LAAC to DOACs	Amulet™
Est. 2028	ELAPSE	Comparing LAAC plus OAC vs. OAC alone in recurrent stroke despite therapy	—
Est. 2029	LAAOS-IV	Comparing LAAC plus OAC vs. OAC alone in AF patients	WATCHMAN FLX™ PRO

*Abbreviations:* AF, atrial fibrillation; CABG, coronary artery bypass grafting; DAPT, dual antiplatelet therapy; DOAC, direct oral anticoagulant; LAA, left atrial appendage; LAAC, left atrial appendage closure; NVAF, non-valvular atrial fibrillation; OAC, oral anticoagulation; RCT, randomized controlled trial; SAPT, single antiplatelet therapy.

**Table 2:** Timeline of Main Findings of the Randomized Controlled Trials and Observational Registries on Left Atrial Appendage Closure

Author	Study	Device	Follow-up Length	Acute Success	Periprocedural Complications (Up to 7 Days)	Peri-device Leak	Device-related Thrombus	Device Embolization	Stroke/Systemic Embolism	Bleeding	Mortality
Holmes et al. (2009)^14^	PROTECT AF	WATCHMAN™ 2.5	2 years	91%	Pericardial tamponade, 4.3% Pericardial effusion, 0.9% Procedure-related stroke, 1.15% Device embolization, 0.6%	8% at 12 months	NA	0.6%	Ischemic stroke, LAAC 1.3 events per 100 patient-years vs. warfarin 1.6 events per 100 patient-years Hemorrhagic stroke, LAAC 0.2% vs. warfarin 2.5%	LAAC 3.5% vs. warfarin 4.1%	LAAC 3.0% vs. warfarin 3.1% at 1 year LAAC 5.9% vs. warfarin 9.1% at 2 years
Reddy et al. (2013)^39^	ASAP	WATCHMAN™ 2.5	1 year	94.7%	Overall, 8.7% Pericardial tamponade, 1.3% Pericardial effusion, 2.0% Procedure-related stroke, 0.7% Device embolization, 1.3% Femoral pseudoaneurysm, 0.7% Femoral hematoma/bleeding, 1.3%	NA	4%	1.3%	Ischemic stroke, 1.7% Hemorrhagic stroke, 0.6%	NA	5% at 12 months
Holmes et al. (2014)^16^	PREVAIL	WATCHMAN™ 2.5	1 year	95.1%	Pericardial tamponade, 1.9% Procedure-related stroke, 0.37% Major bleeding, 0.4% Device embolization, 0.7%	NA	NA	0.7%	Ischemic stroke, LAAC 1.9% vs. control 0.7% Hemorrhagic stroke, LAAC 0.4% vs. control 0% Systemic embolism, LAAC 0.4% vs. control 0%	0.4%	LAAC 2.6% vs. control 2.2% at 12 months
Reddy et al. (2017)^38^	5-year outcomes (PROTECT AF & PREVAIL)	WATCHMAN™ 2.5	5 years	NA	NA	NA	NA	NA	Overall, LAAC 1.7% vs. control 1.8%; *P* = .87 Ischemic stroke or systemic embolism, LAAC 1.6% vs. control 0.95%; *P* = .08 Hemorrhagic stroke, LAAC 0.17% vs. control 0.87%; *P* = .0022 Ischemic stroke or systemic embolism >7 days, LAAC 1.3% vs. control 0.95%; *P* = .28	Major bleeding, LAAC 3.1% vs. control 3.5%; *P* = .60 Major bleeding, non–procedure-related, LAAC 1.7% vs. control 3.6%; *P* = .0003	All-cause, LAAC 3.6% for LAAC group vs. control 4.9% or control group; *P* = .035 CV/unexplained, LAAC 1.3% vs. control 2.2%; *P* = .027
Landmesser et al. (2018)^26^	Global Amulet observational registry	Amulet™	1 year	99.3%	Pericardial effusion/tamponade, 1.65% Procedure-related death, 0.28%	1.6% (flow >3 mm)	1.7%	NA	Ischemic stroke, 2.9% Transientischemic attack, 0.9% Systemic embolism, 0%	Major bleeding, 10.3%	All-cause, 8.4% CV, 5%
Boersma et al. (2017)^47^	EWOLUTION	WATCHMAN™ 2.5	732 days	98.5%	Pericardial tamponade, 0.29% Pericardial effusion, 0.39% Procedure-related stroke, 0.10% Device embolization, 0.2%	0.7% (flow >5 mm)	4.1%	0.2%	All stroke, 1.3/100 patient-years Composite of TIA/stroke, 2.0/100 patient-years	Major bleeding, 2.7/100 patient-years	All-cause, 15.9% CV, 4.5% Bleeding-related, 1% Unexplained, 2.9%
Holmes et al. (2019)^15^	CAP and CAP2 registries	WATCHMAN™ 2.5	50.1–50.3 months	94.3%	Pericardial tamponade, CAP 1.4%; CAP2 1.9% Pericardial effusion, CAP 0.9%; CAP2 0.5% Procedure-related stroke, CAP 0%; CAP2 0.35% Device embolization, CAP 0.2%; CAP2 0%	NA	CAP 2.6%, CAP2 3.9%	CAP 0.2%, CAP2 0%	Overall, CAP 1.48%; CAP2 2.25% Ischemic stroke, CAP 1.30%; CAP2 2.20% Hemorrhagic stroke, CAP 0.17%; CAP2 0.09% Systemic embolism, CAP 0.04%; CAP2 0.09%	NA	All-cause, CAP 4.27% vs. CAP2 6.24% CV/unexplained, CAP 1.69% vs. CAP2 2.92%
Freeman et al. (2020)^49^	NCDR LAA occlusion registry	WATCHMAN™ 2.5	NA	98.3%	Overall major adverse events, 2.16% Cardiac arrest, 0.24% Myocardial infarction, 0.04% Pericardial tamponade, 1.39 Major vascular complications, 0.15 Device embolization, 0.07%	NA	NA	0.07%	Ischemic stroke, 0.12% Hemorrhagic stroke, 0.01% Undetermined stroke, 0.01% TIA, 0.04% Intracranial hemorrhage, 0.01%	1.25%	All-cause, 0.19%
Osmancik et al. (2020)^50^	PRAGUE-17	WATCHMAN™ 2.5/FLX™ vs. Amulet™	21.1 months	NA	Overall, 2.00% Pericardial effusion, 1% Procedure-related death: 1% Major vascular complications, 1% Device embolization, 0.5%	NA	NA	0.5%	Overall, DOAC 2.57% vs. LAAC 2.60% Ischemic stroke/TIA, DOAC 2.28% vs. LAAC 2.60% Systemic embolism, DOAC 0.29% vs. LAAC 0%	DOAC 7.42% vs. LAAC 5.50%	CV, DOAC 4.28% vs. LAAC 3.18%
Kar et al. (2021)^17^	PINNACLE FLX	WATCHMAN FLX™	1 year	98.8%	0.5%	17.2% at 45 days; 10.5% at 12 months	1.86%	0%	All stroke, 2.6% (no hemorrhagic stroke) TIA, 0.3% Systemic embolism, 0.3%	7.9%	6.6%
Lakkireddy et al. (2021)^27^	Amulet IDE	Amulet™ vs. WATCHMAN™ 2.5	1 year	Amulet 98.4% vs. WATCHMAN 96.4%	Amulet 4.5% vs. WATCHMAN 2.5%; *P* = .02; largely related to more frequent pericardial effusion and device embolization	Amulet 1.1% vs. WATCHMAN 3.2% (*P* = .003)	Amulet 3.3% vs. WATCHMAN 4.5%; *P* > .05	NA	Ischemic stroke at 12 months, Amulet 2.5% vs. WATCHMAN 2.7%; *P* > .05 Hemorrhagic stroke at 12 months, Amulet 0.3% vs. WATCHMAN 0.7%; *P* > .05 Systemic embolism, Amulet 0.3% vs. WATCHMAN 0.2%; *P* > .05 TIA at 12 months, Amulet 1.6% vs. WATCHMAN 1.4%; *P* > .05	Amulet 11.6% vs. WATCHMAN 12.3%; *P* > .05	Amulet 3.9% vs. WATCHMAN 5.1%; *P* > .05
Kapadia et al. (2024)^22^	SURPASS	WATCHMAN FLX™	1 year	97.5%	Overall, 1.3% Procedure-related death, 0.09 Procedure-related stroke or systemic thromboembolism, 0.09% Major bleeding, 1.0% Major vascular complication, 0.12% Myocardial infarction, 0.02% Pericardial tamponade, 0.39% Device embolism, 0.03% Air embolism, 0.03%	12.8%	0.44%	0.04%	All stroke, 1.5% Ischemic stroke, 1.2%	6.4%	8.2%
Galea et al. (2025)^33^	SWISS-APERO 3-year outcomes	Amulet™ vs. WATCHMAN FLX™ (>80%)/2.5	3 years	NA	Amulet 2.7% vs. WATCHMAN 0.9%; *P* = .341	Amulet 19% vs. WATCHMAN 20% at CCTA; 13.7% vs. 27% at TEE (45 days); 28.6% vs. 27.5% at CCTA (13 months)	Amulet 3.6% vs. WATCHMAN 6.4%	NA	All stroke, Amulet 5% vs. WATCHMAN 9.4 %; *P* = .289 Ischemic stroke, Amulet 5% vs. WATCHMAN 7.2%; *P* = .566 Hemorrhagic stroke, Amulet 0% vs. WATCHMAN 0.9%; *P* = .498 TIA, Amulet 1.1% vs. WATCHMAN 4.5%; *P* = .215 Systemic embolism, Amulet 0% vs. WATCHMAN 2.2%; *P* = .247	Any bleeding, Amulet 45.1% vs. WATCHMAN 43.2%; *P* = .414 Minor bleeding, Amulet 37.4% vs. WATCHMAN 29.7%; *P* = .225 Major bleeding, Amulet 16.5% vs. WATCHMAN 18%; *P* = .938	All-cause, Amulet 27% vs. WATCHMAN 31%; *P* = .544 CV, Amulet 13.5% vs. WATCHMAN 23.4%; *P* = .101
Wazni et al. (2025)^52^	OPTION	WATCHMAN FLX™	36 months	98.8%	Overall, 2.8%	20.3%	1.9%	NA	Ischemic stroke, LAAC 1.2% vs. control 1.3% Hemorrhagic stroke, 0.4% in each group Systemic embolism, LAAC 0.3% vs. control 0.1%	Major bleeding, LAAC 3.9% vs. control 5.0% Major bleeding, non–procedure-related, LAAC 8.5% vs. control 18.1%; *P* < .001	All-cause, LAAC 3.8% vs. control 4.5%

*Abbreviations:* CCTA, cardiovascular computed tomography angiography; CV, cardiovascular; DOAC, direct oral anticoagulant; LAAC, left atrial appendage closure; NA, not available; TEE, transesophageal echocardiography; TIA, transient ischemic attack.

## Evolution of left atrial appendage closure: from surgery to catheter-based approaches

The first description of a stroke-preventive local therapy involving LAA exclusion or isolation was provided by Madden in 1949.^6^ This study involved resection of the left auricular appendix in two relatively young patients as a prophylactic measure against recurrent arterial emboli, following the intraoperative finding of thrombi within the auricular appendix. This early feasibility experience was later validated in a larger surgical cohort.^7^ Blackshear and Odell subsequently provided additional data supporting the link between AF-related stroke and LAA thrombus formation.^2^ In a retrospective surgical cohort, they demonstrated that >90% of patients referred for cardiac surgery with NVAF and a history of stroke had thrombi located within the LAA. In contrast, this was true for only about 60% of patients with valvular AF. In a more recent prospective cohort of 1450 patients referred for external cardioversion, Cresti et al. found that >99% of thrombi detected via transesophageal echocardiography (TEE) were located in the LAA.^8^ The LAA has therefore rightfully earned the title of “most lethal human attachment.”^7^

The first large, prospective RCT evaluating the impact of LAAC on stroke incidence was LAAOS (“Left Atrial Appendage Occlusion Study”).^9^ This study enrolled patients who underwent surgical LAAC (S-LAAC) using either epicardial suture or stapling techniques. While the trial did not demonstrate a significant reduction in post-procedural stroke incidence in the intervention group compared to controls, it did establish the feasibility and safety of S-LAAC. Moreover, it highlighted the need for more effective LAAC strategies and laid the groundwork for future trials. The pivotal LAAOS III trial later provided conclusive evidence that LAA occlusion during cardiac surgery reduces the risk of stroke and systemic embolism (SE) by 33%, even among patients receiving OAC.^10^ Notably, this benefit was achieved without an increase in major bleeding or surgical complications, confirming both the efficacy and safety of the intervention. These findings have prompted changes in clinical practice guidelines, including the recommendation for routine S-LAAC in patients with AF undergoing cardiac surgery and in those at high risk of thromboembolism.^3,4^

## Evolution of percutaneous left atrial appendage closure devices

After the initial success of S-LAAC approaches, minimally invasive catheter-based solutions began to emerge. The first of these were the PLAATO system (eV3, Inc., Plymouth, MA, USA) and the WATCHMAN™ device (Boston Scientific, Marlborough, MA, USA).^11,12^ The PLAATO system was the first device specifically designed for LAAC to be approved for implantation in humans. It consisted of a self-expandable nitinol cage covered with an occlusive expanded polytetrafluoroethylene (PTFE).^13^ The PLAATO (“Percutaneous Left Atrial Appendage Transcatheter Occlusion”) study, the first multicenter trial on LAAC, demonstrated a high procedural success rate (approximately 90%) and acceptable safety profile, with a 10% incidence of residual leak on 2-month follow-up TEE imaging. Despite these promising results, the PLAATO device was eventually discontinued due to financial and commercial constraints. Following its discontinuation, research efforts rapidly shifted toward developing safer, more effective, and user-friendly transcatheter solutions. Among the various technologies explored, two device families, WATCHMAN™ and Amplatzer™, emerged as the most widely adopted and clinically validated options for LAAC.

## Endocardial approach

### WATCHMAN™ device series

The WATCHMAN™ device was the first percutaneous LAAC system to gain widespread clinical use. Structurally, the original WATCHMAN™ 2.5 device featured a parachute-shaped, self-expanding nitinol frame covered with a polyethylene terephthalate (PET) membrane designed to promote endothelialization and prevent thrombus embolization.^12^ Its safety and efficacy were first evaluated in the landmark PROTECT AF (“Watchman Left Atrial Appendage Closure Technology for Embolic Protection in Patients With Atrial Fibrillation”) trial,^14^ which demonstrated non-inferiority to warfarin for stroke prevention in patients with NVAF. However, there was a higher initial rate of periprocedural complications, such as pericardial effusion and air embolism (see the discussion on LAAC RCTs). Subsequent data, including those from the PREVAIL (“Prospective Randomized Evaluation of the Watchman LAA Closure Device in Patients with Atrial Fibrillation versus Long-term Warfarin Therapy”) trial and the CAP/CAP2 (“Continued Access to PROTECT AF and PREVAIL,” respectively) registries,^15,16^ confirmed the feasibility of the procedure, with improved safety outcomes over time, reflecting an increase in operator experience and refinement in technique. The WATCHMAN FLX™ device represented the next generation of the WATCHMAN™ device series, incorporating several key design improvements over the previous WATCHMAN™ 2.5 model.^17^ Notably, the FLX™ features a greater number of open-frame cells, enhancing device conformability, and a 10%–20% reduction in length, allowing for implantation in shallower LAAs (requiring only 50% of the device size in depth). Compared to WATCHMAN™ 2.5, the number of fixation anchors was increased to 18 J-shaped anchors arranged in two rows, improving stability. The FLX also introduced a closed, atraumatic distal end with a fluoroscopic marker, facilitating safer advancement into the LAA, particularly when partially deployed in a “ball” configuration, reducing the risk of distal perforation. Further enhancements included extended polyester fabric coverage to minimize peri-device leaks (PDLs) and a recessed delivery cable screw to reduce the risk of device-related thrombus (DRT).^18^

The pivotal PINNACLE FLX (“Protection Against Embolism for Non-valvular AF Patients: Investigational Device Evaluation of the Watchman FLX LAA Closure Technology”) trial provided the first prospective evaluation of the WATCHMAN FLX™ device.^17^ In this study, the primary effectiveness endpoint of adequate LAAC was met in 100% of evaluable patients at both 45 days and 12 months. The mean PDL was 0.03 ± 0.22 mm at implantation and 0.57 ± 1.26 mm at 45 days, and no patient had a residual jet of >5 mm at 12 months. In addition 90% of patients had no detectable PDL on core-laboratory assessment at that time point; conversely, at 1 year, the same finding was observed in up to 40.9% and 32.1% of patients enrolled in the PROTECT AF and PREVAIL studies, respectively. This is noteworthy given that recent evidence suggests an increased stroke risk in patients with incomplete LAAC due to small (0–5 mm) PDLs.^19,20^ At 12 months, the Kaplan–Meier estimated incidence rates of all-cause mortality and any stroke were 6.6% and 2.6%, respectively, with no hemorrhagic strokes reported and a single SE (0.3%). Pericardial effusion requiring intervention occurred in 1.0% of patients over 12 months. DRT was detected in seven patients within the first year, most frequently during scheduled follow-up TEE, and only two of these cases were associated with a clinically apparent embolic event. This rate compares favorably with the 3.7% of patients in whom DRT was reported before.^21^ These data established WATCHMAN FLX™ as an effective device for achieving durable LAAC with low rates of serious procedure- and device-related complications in a controlled trial setting.

These results were confirmed in routine practice by a sub-analysis of the LAAC registry, SURPASS (“Watchman FLX Device Surveillance Post-approval Analysis Plan”), which reported a 97.5% implantation success rate, very low rates of serious in-hospital complications, and excellent sealing performance: a PDL >5 mm was almost absent, and approximately 99%–100% of patients met the definition of effective closure (jet ≤ 5 mm) at early follow-up. At 1 year, all-cause mortality was around 8%, with any stroke in 1.5% and ischemic stroke in 1.2%, along with major bleeding in about 6%, indicating that the safety and effectiveness observed in the trial are largely reproduced in a broad, unselected clinical population.^22^ For these reasons, the WATCHMAN FLX™ device has effectively replaced the previous-generation device as the preferred option for LAAC in current clinical practice. WATCHMAN FLX™ PRO, the latest-generation device in the WATCHMAN™ series, was designed to further reduce DRT through the use of a novel polymer coating that promotes rapid endothelialization.

The evidence for WATCHMAN FLX™ PRO currently consists of early, industry-sponsored registry data from the SURPASS Pro analysis of the NCDR (“National Cardiovascular Data Registry”) LAAO Registry, with high procedural success (≈97%), a very low 7-day key safety endpoint rate (≈0.20%), and excellent sealing performance (≈88% complete seal and 98% clinically relevant closure ≤3 mm at 45 days) but only short-term follow-up and no randomized or long-term comparative data to date. Therefore, although WATCHMAN FLX™ PRO shows promising early outcomes, its evidence base remains less mature than that of WATCHMAN FLX™.^23^

### Amplatzer™ family: Amplatzer™ Cardiac Plug and Amulet™

In parallel to the WATCHMAN™ series, the Amplatzer™ family of LAAC devices (originally developed by St. Jude Medical and later Abbott) offers a distinct dual-seal design for LAA occlusion. The first generation of Amplatzer™ devices included the Amplatzer™ Cardiac Plug (ACP), a self-expanding nitinol mesh structure featuring two polyester patches, one attached to the distal lobe and the other to the proximal disc, connected by an articulated waist. The lobe was typically implanted approximately 10 mm inside the LAA body and anchored by a variable pair of stabilizing wires, depending on the device size. Notably, unlike the WATCHMAN™ device, the ACP did not require OAC following implantation. Instead, patients were prescribed dual antiplatelet therapy (DAPT) for 1–6 months, followed by single or no antiplatelet therapy. DRT rates were slightly higher than with WATCHMAN™ (4.4% against the 3.86% reported from a pooled analysis of the PROTECT AF, PREVAIL, and CAP1 [“Continued Access to PROTECT AF”] and CAP2 registries), but the simplified antithrombotic regimen made ACP attractive for patients with contraindications to OAC.^21,24^

The first-generation Amplatzer™ devices were discontinued following the release of the second-generation model, ACP2, commercially known as the Amplatzer™ Amulet™. The design of Amulet™ has been improved in comparison to its predecessor to address issues such as PDLs, device embolization, and complex LAA anatomies. These changes included: (1) a longer distal lobe for deeper anchoring; (2) an increased number and stiffness of anchoring hooks; (3) wider disc and lobe diameters to accommodate diverse anatomies; and (4) a pre-loaded delivery system for faster, more controlled deployment.^25^

A new iteration of the Amulet™ device (Amulet™ 2/Amplatzer™ 360) is expected to be even safer. Some of the improvements include an atraumatic distal end and a reduced length of the proximal anchoring hooks, with the aim of reducing the incidence of pericardial effusion, tamponade, and collateral vessel perforations. The safety and efficacy of this newer generation of Amplatzer™ device is being explored in the prospective VERITAS study (NCT06707688).

### Comparison between devices: AMULET™ IDE trial and SWISS-APERO trial

The Amulet IDE (“AMPLATZER Amulet Left Atrial Appendage Occluder Investigational Device Exemption [IDE] Trial”) provided the first RCT comparing the efficacy and safety outcomes between WATCHMAN™ and Amplatzer™. In terms of procedure-related efficacy and safety, Amulet™ had a higher rate of successful device-based LAAC compared with WATCHMAN™ 2.5 (98.9% vs. 96.8%; *P* < .001 for non-inferiority; *P* = .003 for superiority), with complete occlusion (defined as no residual jet around the device) achieved in 63% in the Amulet™ group versus 43% in the WATCHMAN™ 2.5 group. Conversely, procedure-related complications were higher for Amulet™ (4.5% vs. 2.5%), with more frequent pericardial effusions and device embolization. Conversely, in a prospective observational study using Amulet™, the reported prevalence rates for device embolization and cardiac perforation were as low as 0.2% and 1.3%, respectively.^26^ This mismatch was mainly attributed by the investigators to the fact that the operators who took part in the Amulet™ IDE trial were from US sites with no previous Amulet™ experience. Furthermore, the prevalence of pericardial effusion was higher among patients receiving Amulet™ who were discharged on OAC. The difference may have been driven by the intrinsically higher bleeding risk of the Amulet™ group. The less-intensive antithrombotic therapy required for LAAC with Amulet™ in real-world practice may mitigate the risk of late pericardial effusions observed in the Amulet™ IDE study. Notably, even though patients enrolled in the Amulet™ cohort were less likely to be on OAC, DRT rates were similar between groups (3.3% in Amulet™ vs. 4.5% in WATCHMAN). Both devices exhibited comparable ischemic stroke, SE, and cardiovascular death (CVD) rates (*P* < .001 for non-inferiority).^27^ At the long-term 5-year outcome analysis, no differences were observed between the two devices for (1) ischemic stroke or SE; (2) all stroke, SE, or CVD; (3) major bleeding; (4) all-cause death; and (5) CVD. Interestingly, both arms had an annual ischemic stroke rate of 1.6%, 78% lower than the annual stroke risk as predicted by CHA_2_DS_2_-VASc scoring. It was also notable that, although the incidence of stroke was similar in both groups in the Amulet™ IDE study, there were more fatal or disabling strokes among WATCHMAN™ 2.5 recipients compared with Amulet™ recipients (*P* = .03). The investigators suggested that a potential cause may have been the higher prevalence of DRT and PDL among WATCHMAN™ 2.5 patients with stroke compared with Amulet™ patients with stroke.^28^ It should be noted that, in this trial, only the first-generation WATCHMAN™ device was available for the comparison.

Since then, second- and third-generation WATCHMAN™ devices have been commercially released, with multiple design changes, which have been shown to reduce the risk of pericardial effusion, major bleeding, PDL, and likely DRT.^29^ In the SWISS-APERO (“Comparison of Amplatzer Amulet and Watchman Device in Patients Undergoing Left Atrial Appendage Closure”) RCT, the efficacy and the safety profiles of WATCHMAN™ 2.5/FLX™ and Amulet™ devices were compared for the first time. At the 45-day efficacy analysis, both groups were assessed for LAA patency using cardiac computed tomography angiography (CCTA). The patent appendage rate was similar between the two groups. However, the underlying mechanisms significantly differed between the two arms.^30^ When assessed by TEE, PDL rates were twofold higher with WATCHMAN™ than with Amulet™ (27.5% vs. 13.7%; *P* = .020). When the leaks detected by CCTA were categorized by mechanism, WATCHMAN™ showed a lower rate of intra-device leaks compared to Amulet™ (23% vs. 44.8%; *P* = .001), but a higher rate of mixed leaks (14% vs. 3.8%; *P* = .01). Unlike CCTA, TEE detects LAA leaks through direct visualization of high-velocity flows adjacent to the device lobe, regardless of whether the flow extends along the entire lobe length or only a portion of it.^31^ The leaks identified by TEE therefore would largely correspond to the mixed leaks and PDLs detected by CCTA.

At the 13-month analysis, LAA patency was observed in 53.6% with Amulet™ versus 48.8% with WATCHMAN™ 2.5/FLX™ (risk ratio, 1.10; 95% confidence interval [CI], 0.81–1.48; *P* = .537). Conversely, in the Amulet™ IDE trial, the rate of LAA complete occlusion at the 12-month TEE was higher with Amulet™ compared with WATCHMAN™ 2.5 (63.2% vs. 53.1%; *P* < .01).^27^

It is likely that differences in the type of devices used as well as the different primary imaging modalities used to assess LAA patency may partially explain the different LAA patency outcomes observed in these two trials. On the other hand, similar to the Amulet™ IDE trial, the SWISS-APERO trial did not show any significant differences between Amulet™ and WATCHMAN™ in CVD, ischemic stroke and SE, cerebrovascular events, or bleeding.^32^

At the 3-year analysis, DRT and PDL rates detected at CCTA did not differ significantly between the two groups (*P* > .05). In addition, no significant associations were found between DRT or PDL and ischemic events. The Amulet™ group exhibited a lower incidence of CVD, stroke, transient ischemic attack (TIA), or SE compared with the WATCHMAN™ 2.5/FLX™ group (18.2% vs. 31%; hazard ratio [HR], 0.58; 95% CI, 0.33–1.03; *P* = .062). This trend was driven by numerically lower rates of CVD (13.5% vs. 23.4%), stroke (5.0% vs. 9.4%), and TIA (1.1% vs. 4.5%). When periprocedural events occurring within 7 days were censored, the Amulet™ arm showed a significantly lower rate of the composite outcome compared to the WATCHMAN FLX™ group (HR, 0.50; 95% CI, 0.28–0.92; *P* = .026).^33^ However, the study was not powered for this clinical endpoint, and further studies are required to confirm potential device-related differences in clinical outcomes.

## Endo-epicardial approach

### LARIAT

The LARIAT device (SentreHEART, Redwood City, CA, USA) is a percutaneous epicardial suture–based ligation system for LAAC that avoids the deployment of a long-term implant inside the heart. It is placed through a combination of transseptal (endocardial) and pericardial access. Two magnet-tipped guidewires, one introduced via the atrium and the other through the pericardial space, are aligned to allow delivery of a pre-tied polyester snare around the base of the LAA. Once deployed, the snare is cinched externally, ligating the LAA from the outside and promoting natural endothelialization without the use of a foreign material. The system includes a pericardial sheath, an occlusion balloon to shape the LAA during positioning, and a non-absorbable suture loop. Over time, the system has evolved to improve safety, anatomical compatibility, and operator control. The second-generation LARIAT+ improved upon the design, with an increased snare size (up to 45 mm), a fluoroscopic orientation marker, and a braided shaft for better torque control.^34^ The third-generation LARIAT RS included faster, more intuitive deployment and improved magnet alignment. In this version, the use of a pigtail catheter to fill and direct the LAA endocardially is supposed to improve procedural safety and control.^35^ The device’s implant-free design eliminates potential DRT and minimizes long-term foreign body exposure. In the first human study of the LARIAT device, a 96% acute procedural success rate was achieved, with a 98% rate of complete LAAC at 12-month follow-up. Most residual leaks were ≤3 mm, and complications were limited. These were mainly related to epicardial access, and no device-related deaths or embolic events were reported.^36^ However, in the aMAZE (“LAA Ligation Adjunctive to PVI for Persistent or Longstanding Persistent Atrial Fibrillation”) clinical trial, complete closure of the LAA with the LARIAT+/RS device was achieved in 84% of patients at 12 months, with a closure rate of 99% when including cases with residual communication of ≤5 mm.^35^

## Clinical evidence in left atrial appendage closure

The evolution of LAAC technology has been closely paralleled by a robust body of clinical research aimed at establishing its safety, efficacy, and appropriate patient selection. Initial trials focused on proving non-inferiority to warfarin, the standard of care at the time these devices first emerged, while more recent studies have begun to explore comparisons with direct oral anticoagulants (DOACs) and investigations in high-bleeding-risk populations.

### Warfarin era: PROTECT AF, PREVAIL, and long-term outcomes

In the PROTECT AF trial, LAAC with the WATCHMAN™ device was shown to be feasible and non-inferior to OAC with warfarin for the prevention of embolic stroke in patients with NVAF for the first time.^14^ However, the study registered a higher-than-expected rate of complications, mainly pericardial effusions and air embolism–related strokes. Additionally, a potential population selection bias may have played a role, given the low median CHADS_2_ score in this population.

To address these early safety concerns and methodological issues, WATCHMAN™ investigators re-evaluated the device in the PREVAIL trial. In this study, both the acute procedural success rate and the safety profile improved, aligning more closely with the outcomes reported in the later phase of the PROTECT AF study and the CAP1 registry.^37^ The 7-day procedure-related complication rate (defined as a composite of cardiac perforation, pericardial effusion with tamponade, ischemic stroke, device embolization, and other vascular complications) was 4.2% compared to 8.7% in the PROTECT trial (*P* = .004). In addition, a decrease in the rate of pericardial effusions requiring surgical intervention (from 1.6% to 0.4%; *P* = .027) and of procedural and device-related strokes (from 1.1% to 0.4%, *P* = .007) was detected.^17^ Nevertheless, the trial failed to meet the primary efficacy endpoint due to the low event rate. The combined 5-year outcome analysis of the PREVAIL and PROTECT AF trials revealed a comparable risk of all stroke with LAAC compared to warfarin (HR, 0.96; 95% CI, 0.60–1.54; *P* = .87) but significant reductions in hemorrhagic stroke (HR, 0.45; 95% CI, 0.21–0.94; *P* = .034), cardiovascular mortality (HR, 0.59; 95% CI, 0.37–0.94; *P* = .027) and all-cause mortality (HR, 0.73; 95% CI, 0.54–0.98; *P* = .035, respectively).^38^ Moreover, after excluding procedure-related events, significantly less bleeding was observed in the LAAC group (HR, 0.48; 95% CI, 0.32–0.71; *P* = .0003). However, these large RCTs lacked a comparison with DOACs and did not include patients deemed unsuitable for long-term OAC therapy, the group most likely to benefit from the LAAC procedure. Additionally, it is important to note that all patients undergoing LAAC received short-term OAC therapy for at least 6 weeks post-procedure. While this period is considered necessary for complete endothelialization of the device, it may not be feasible for patients at high risk of bleeding.

The feasibility of a non-OAC transitional pharmacological regimen post-LAAC with WATCHMAN™ was explored in the observational ASAP (“ASA Plavix Feasibility Study with Watchman Left Atrial Appendage Closure Technology”) study, in which consecutive AF patients ineligible for warfarin were treated with LAAC followed by DAPT.^39^ This registry enrolled AF patients with an average CHADS_2_ score of 2.8 points, corresponding to an expected annual ischemic stroke rate of 7.3% if treated with aspirin alone.^40^ However, the observed stroke rate was only 1.7%, representing a 77% reduction compared to the expected rate. Patients in the ASAP study also received a 6-month treatment with clopidogrel, a drug shown in the ACTIVE A (“Atrial Fibrillation Clopidogrel Trial with Irbesartan for Prevention of Vascular Events”) trial to reduce the risk of stroke by 32% compared to aspirin alone.^41^ Even after adjusting the expected stroke rate to 5.0% to account for this benefit (although the patients in the ASAP registry did not receive long-term clopidogrel), the observed 1.7% rate still reflected a 64% reduction, indicating a greater-than-expected protective effect. The annualized rates of all-cause stroke and ischemic stroke reported for the ASAP study were also similar to the ones reported in the PROTECT AF trial (2.3% and 1.7%, respectively). Despite the nonrandomized design and absence of a control group, the results supported the feasibility of a DAPT-based transitional strategy without an apparent increase in stroke risk.

### Direct oral anticoagulant era

As DOACs became the new standard of care,^42–44^ the more recent LAAC trials aim to test whether a non-pharmacological stroke-management strategy could remain a viable alternative. According to the current American and European guidelines, LAAC may be considered for AF patients with contraindications to long-term OAC, such as history of intracranial hemorrhage, recurrent mucosal (ie, gastrointestinal [GI], pulmonary, or genitourinary) bleeding, active malignancy associated with a high bleeding risk, documented bleeding diatheses, and chronic kidney disease (CKD). In addition, clinical scenarios such as frequent traumatic falls due to non-reversible causes in frail patients may be recognized as a valid reason to consider LAAC in place of anticoagulation.^3–5,45,46^ Based on the 2023 American College of Cardiology (ACC)/American Heart Association (AHA), 2024 European Society of Cardiology (ESC), and 2025 Society for Cardiovascular Angiography & Interventions (SCAI)/Heart Rhythm Society (HRS) recommendations, a practical, guideline-based algorithm for the selection of candidates for surgical or percutaneous LAAC is summarized in **Figure 1**. The flowchart integrates stroke risk (CHA_2_DS_2_-VASc), OAC contraindications or intolerance, high bleeding risk, and post-ablation status to guide when LAAC may be considered as an alternative or adjunct to OAC.

**Figure 1:**
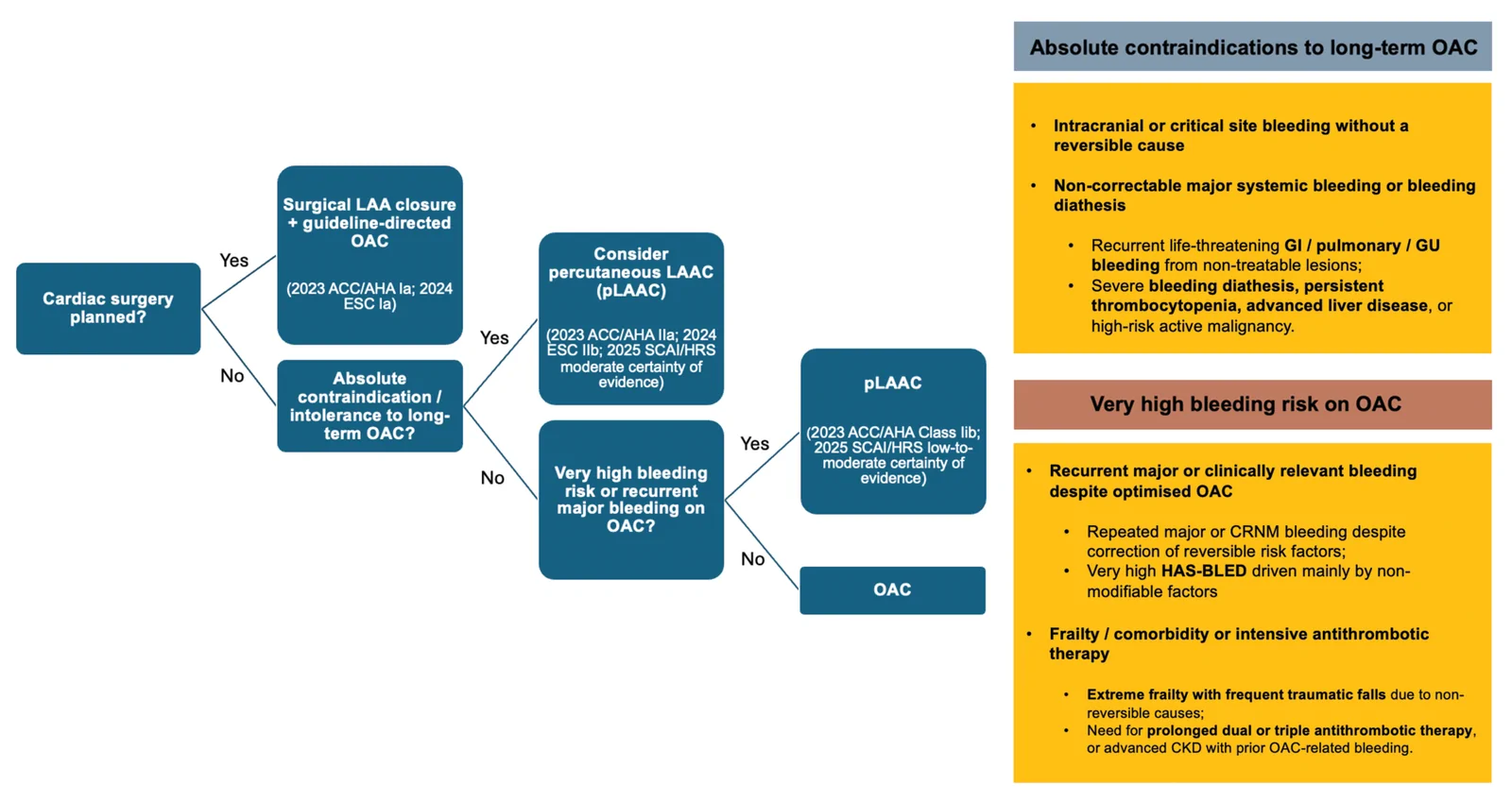
Proposed decision-making algorithm for left atrial appendage closure (LAAC). The figure summarizes the clinical pathway for considering surgical or percutaneous LAAC in patients with absolute contraindications to long-term oral anticoagulation (OAC) or very high/recurrent bleeding risk despite OAC. Abbreviations: ACC, American College of Cardiology; AHA, American Heart Association; CKD, chronic kidney disease; CRNM, clinically relevant non-major; ESC, European Society of Cardiology; GI, gastrointestinal; GU, genitourinary; HRS, Heart Rhythm Society; SCAI, Society for Cardiovascular Angiography & Interventions.

Several pieces of evidence support the use of LAAC in routine clinical practice **(Table 3)**. Aggregate data from large-scale registries (ie, the EWOLUTION [“Evaluating Real-life Clinical Outcomes in Atrial Fibrillation Patients Receiving the Watchman Left Atrial Appendage Closure Technology”] registry for WATCHMAN™ devices and the global Amulet™ study) continue to provide optimal data on the efficacy and the safety profile of LAAC **(Table 2)**. Recent studies also reveal improved outcomes, likely attributable to enhanced procedural planning, advancements in device design and surface characteristics, and greater operator experience.^26,47–49^ In the PRAGUE-17 trial (“Percutaneous Left Atrial Appendage Closure vs. Novel Anticoagulation Agents in Atrial Fibrillation”), AF patients with a history of cardioembolic event and a clinically relevant bleeding risk (CHA_2_DS_2_-VASc score ≥ 3 points and a HAS-BLED score ≥ 2 points) were randomized to receive LAAC (using Amulet™, WATCHMAN™ 2.5, and WATCHMAN FLX™ devices) or DOAC (95% of patients received apixaban). The results established the non-inferiority of LAAC compared to OAC for the primary efficacy composite endpoint of stroke, TIA, SE, CVD, major or non-major clinically relevant bleeding, and procedure- or device-related complications (*P* = .0004)^50^ and were later confirmed at the 4-year analysis.^51^ The OPTION (“Comparison of Anticoagulation with Left Atrial Appendage Closure After AF Ablation”) trial recently assessed the safety and efficacy of LAAC in patients with moderate-to-high stroke risk undergoing catheter ablation for AF. Participants were randomized to receive LAAC or standard OAC following the ablation procedure. The results showed a superiority for the primary endpoint of major bleeding or minor clinically relevant bleeding events with LAAC (*P* < .001 for superiority) and non-inferiority for the composite endpoint of all-cause mortality, stroke, or SE (*P* < .001) at 36 months. These findings support the potential value of combining AF ablation with LAAC to reduce stroke risk while enabling early discontinuation of anticoagulation, thereby improving the overall safety of stroke-prevention strategies.^52^

**Table 3:** Timeline of Comparative RCT Evidence in LAAC: Antithrombotic Therapy and Clinical Outcomes

Author	Study	Device	Population	Antithrombotic Therapy	Primary Efficacy Endpoint	Safety Endpoint/Adverse Events
Holmes et al. (2009)^14^	PROTECT AF	WATCHMAN™ 2.5 vs. warfarin	Age, 71.7 ± 8.8 vs. 72.7 ± 9.2 years CHADS_2_ points, 2.2 HAS-BLED points, NA	Warfarin + aspirin (81–325 mg) for 45 days, aspirin + clopidogrel (75 mg) for 6 months, aspirin indefinitely	Composite of stroke, systemic embolism, and CV/unexplained death: LAAC 3.0 per 100 patient-years vs. control 4.9 per 100 patient-years (risk ratio, 0.62; 95% CI, 0.35–1.25)	LAAC 7.4 per 100 patient-years vs. control 4.4 per 100 patient-years
Holmes et al. (2014)^16^	PREVAIL	WATCHMAN™ 2.5 vs. warfarin	Age, 74.0 ± 7.4 vs. 74.9 ± 7.2 years CHADS_2_ points, 2.6 HAS-BLED points, NA	Warfarin + aspirin (81 mg) for 45 days, aspirin + clopidogrel (75 mg) for 6 months, aspirin indefinitely	Composite of stroke, systemic embolism, and CV/unexplained death: LAAC 0.064 vs. control 0.063 (risk ratio, 1.07; 95% CI, 0.57–.89) Stroke or systemic embolism >7 days post-randomization: LAAC 0.025 vs. control 0.020 (risk difference, 0.005; 95% CI, −0.019 to 0.027)	4.2%
Osmancik et al. (2020)^50^	PRAGUE-17	WATCHMAN™ 2.5/FLX™/Amulet™ vs. DOAC	Age, 73.2 ± 7.2 vs. 73.4 ± 6.7 years CHA_2_DS_2_-VASc points, 4.7 ± 1.5 HAS-BLED points, 3.0 ± 0.9	LAAC: aspirin (100 mg) + clopidogrel (75 mg) for 3 months, aspirin indefinitely NOAC: 95.5% apixaban	Composite of stroke, TIA, systemic embolism, CV death, major or non-major clinically relevant bleeding, and procedure-/device-related complications: LAAC 10.99% vs. DOAC 13.42% (hazard ratio, 0.84; 95% CI, 0.53–1.31; *P* = .44)	4.5%
Lakkireddy et al. (2021)^27^	Amulet IDE	Amulet™ vs. WATCHMAN™ 2.5	Age, 75.0 ± 7.6 vs. 75.1 ± 7.6 years CHA_2_DS_2_-VASc points, 4.5 ± 1.3 vs. 4.7 ± 1.4 HAS-BLED points, 3.2 ± 1.0 vs. 3.3 ± 1.0	Amulet: DAPT or NOAC + aspirin for 6 months, aspirin indefinitely; WATCHMAN: warfarin + aspirin for 45 days, DAPT for 6 months, aspirin indefinitely	Composite of ischemic stroke and systemic embolism: 2.8% for both groups (difference, 0.00; 95% CI, − 1.55 to 1.55; *P* = .50)	Composite of procedure-related complications, all-cause death, or major bleeding: Amulet 14.5% vs. WATCHMAN 14.7% (*P* = .47)
Galea et al. (2022)^30^	SWISS-APERO	Amulet™/WATCHMAN FLX™ (>80%)/2.5	Age, 76.5 ± 7.1 vs.77.3 ± 8.4 years CHA_2_DS_2_-VASc points, 4.2 ± 1.4 vs. 4.4 ± 1.4 HAS-BLED points, 3.1 ± 0.8 vs. 3.2 ± 1.0	Aspirin and clopidogrel or OAC for 3 months, aspirin indefinitely	Composite of justified crossover to the nonrandomly allocated device or 45-day peri-device leak: Amulet 67.6% vs. WATCHMAN 70% (risk ratio, 0.97; 95% CI, 0.80–1.16; *P* = .713)	Any periprocedural complication: Amulet 32.4% vs. WATCHMAN 19.1% (*P* = .023) Major procedure-related complications: Amulet 9.0% vs. WATCHMAN 2.7%; *P* = .047)
Wazni et al. (2025)^52^	OPTION	WATCHMAN FLX™	Mean age, 69.7 ± 7.4 vs. 69.4 ± 7.9 years CHA_2_DS_2_-VASc points, 3.5 ± 1.3 HAS-BLED points, 1.2 ± 0.8	OAC and aspirin for 3 months, aspirin indefinitely	Composite of death from any cause, stroke, or systemic embolism: LAAC 5.3% vs. control 5.8% (hazard ratio, 0.91; 95% CI, 0.59–1.39)	Non-procedure-related major bleeding or clinically relevant non-major bleeding: LAAC 8.5% vs. control 18.1% (hazard ratio, 0.44; 95% CI, 0.33–0.59; *P* < .001)

*Abbreviations:* CI, confidence interval; CV, cardiovascular; DAPT, dual antiplatelet therapy; LAAC, left atrial appendage closure; NOAC, novel oral anticoagulant; OAC, oral anticoagulation; RCT, randomized controlled trial.

However, despite these encouraging results, it is important to note that the observed non-inferiority in efficacy might have been influenced by the high rate of sinus rhythm maintenance observed at the 3-year follow-up. In fact, clinical assessment at 36 months showed that >75% of patients were in sinus rhythm, regardless of the treatment group. Nonetheless, the trial also reported a clinical recurrence rate of 65% in both groups, defined as electrical cardioversion, repeat ablation, up-titration of anti-arrhythmic therapy, or documented recurrence of atrial flutter or atrial tachycardia. This apparent discrepancy highlights not only the potential underestimation of AF burden (due to limited rhythm monitoring) but also the possibility that the clinical recurrence rate may overestimate the actual residual stroke risk in patients who are otherwise well-managed. Therefore, while the OPTION trial provides encouraging data supporting LAAC as an adjunct to AF ablation, further large-scale studies with extended rhythm monitoring and longer follow-up are needed to definitively establish the efficacy and safety of this combined approach across diverse patient populations.

The recently reported CLOSURE-AF (“Left Atrial Appendage CLOSURE in Patients with Atrial Fibrillation Compared to Medical Therapy”) trial^53^ provides the second randomized comparison of percutaneous LAAC versus contemporary medical therapy in precisely the population for whom this intervention was originally conceived. In this multicenter German study, 912 patients with AF (median age, 79.1 years; 38.6% women) and both high thromboembolic risk (mean CHA_2_DS_2_-VASc score, 5.2 points) and increased bleeding risk (mean HAS-BLED score of 3.0 points or prior bleeding and/or advanced CKD) were randomized to LAAC (Amplatzer™ Amulet™ or WATCHMAN FLX™) or physician-directed standard care (predominantly DOAC therapy).

Compared with the PRAGUE-17 cohort (mean age, 73.3 years; CHA_2_DS_2_-VASc score, 4.7 points; HAS-BLED, 3.1 points) and especially with the OPTION trial (mean age, 69.6 years; CHA_2_DS_2_-VASc score, 3.5 points; HAS-BLED, 1.2 points), the CLOSURE-AF cohort was substantially older and carried markedly higher absolute risks of both stroke and bleeding.

Over a median follow-up of approximately 3 years, LAAC did not meet the prespecified non-inferiority criterion for the primary composite endpoint of stroke, SE, cardiovascular or unexplained death, or major bleeding. The event rate was 16.83 versus 13.27 per 100 patient-years for LAAC versus standard care, respectively, corresponding to an adjusted HR of 1.28 (95% CI, 1.01–1.62) in favor of medical therapy. Rates of the individual components, including ischemic stroke, were broadly similar between groups, but the trial was underpowered to detect differences in single endpoints. The excess in the composite outcome in the LAAC arm appeared to be largely driven by bleeding, together with a higher rate of cardiovascular/unexplained death. These findings suggest that, in very elderly patients with extreme baseline bleeding risk, the procedural hazards and the burden of post-LAAC antithrombotic therapy may at least partially offset the potential benefit of mechanical stroke prevention. This reinforces the need for individualized regimens and for further trials testing simplified or DOAC-based strategies to preserve the stroke-preventive effect of LAAC without incurring an unacceptably high bleeding burden.

Initial data on the potential role of optimizing post-LAAC therapy also stem from the ANDES (“Short-term Anticoagulation versus Dual Antiplatelet Therapy for Preventing Device Thrombosis Following Left Atrial Appendage Closure”) randomized clinical trial, which compared a short course of DOAC versus DAPT after LAAC. The study showed a lower rate of the composite safety endpoint (death, stroke, bleeding, or treatment-changing DRT) with DOACs, mainly due to fewer bleeding events, without a significant difference in DRT incidence. In this perspective, ongoing randomized studies such as the SIMPLAAFY trial evaluating two monotherapy regimens (aspirin alone or reduced-dose DOAC) versus DAPT after implantation of the WATCHMAN FLX™ PRO device are expected to further clarify whether simplified post-implant regimens can maintain efficacy while mitigating bleeding risk in everyday clinical practice.

The next wave of randomized trials is designed to further refine the role of LAAC across different risk profiles and clinical scenarios. The optimal strategy for AF patients at high thromboembolic and bleeding risk with absolute contraindications to long-term OAC will be addressed in the COMPARE LAAO (“Left Atrial Appendage Occlusion for AF Patients Unable to Use Oral Anticoagulation Therapy”) (NCT04676880) trial, in which patients will be randomized to receive in a 2:1 fashion LAAC followed by DAPT and long-term aspirin or standard medical treatment (single antiplatelet therapy [SAPT] or DAPT).^54^

Conversely, the ongoing CATALYST (“Amplatzer Amulet LAAC vs. DOAC”) (NCT04226547) and CHAMPION-AF (“Watchman FLX vs. DOAC”) (NCT04394546) trials^55^ will help clarify whether percutaneous LAAC might represent an actual alternative to OAC in all subsets of AF patients, irrespective of ischemic or bleeding risk (CHA_2_DS_2_-VASc score of ≥2 points for men or ≥3 points for women in CHAMPION-AF and ≥3 points for both in CATALYST). More recently, the LAAOS-IV trial (NCT05963698) has been enrolling AF patients at high risk of stroke (CHA_2_DS_2_-VASc score of ≥4 points) and eligible for OAC. This trial will assess whether adding the WATCHMAN FLX™ PRO device to standard medical therapy can improve outcomes in AF patients by reducing the incidence of all-cause stroke and systemic thromboembolism. The ELAPSE trial (“Early Closure of Left Atrial Appendage for Patients with Atrial Fibrillation and Ischemic Stroke Despite Anticoagulation Therapy”) (NCT05976685) is focused on addressing the value of added LAAC on top of medical therapy in AF patients who have experienced a stroke despite being on anticoagulant therapy.

### Post-left atrial appendage closure management

Several preclinical studies suggest that completion of the endothelialization process in LAAC recipients may require up to 90 days in humans.^56^

Consequently, dual antithrombotic therapy (DAT) for at least 6 months following LAAC, including both OAC and SAPT or DAPT, has been described and used since the first RCTs. Current guidelines recommend OAC (including DOAC) plus aspirin or DAPT for 45 days, followed by 4.5 months of DAPT and then aspirin alone.^5,46^

However, antithrombotic strategies used in daily clinical practice often diverge from these recommendations, as most patients referred to LAAC are elderly, have a high bleeding risk, and are deemed ineligible even for short-term OAC.^57^

Moreover, specific individual circumstances, such as a history of major bleeding or renal dysfunction, frequently require a personalized approach, and the evidence available so far suggests that antithrombotic therapy after LAAC should be tailored based on several parameters, including patient characteristics and procedural outcomes. Current European device labeling recommends the use of DAPT for 3 months in patients in whom the standard PROTECT AF post-implantation regimen cannot be followed after WATCHMAN™ or ACP/Amulet™ implantation.^46^

Several reports suggest that DAPT may increase the bleeding risk, calling its net benefit into question, especially in those with prohibitive hemorrhagic risk.^58,59^

In the Amulet Observational Study, the major bleeding rate at 1-year follow-up was 10.1%, with most bleeding events occurring within 3 months after LAAC in patients discharged on DAPT.^60^

Søndergaard et al. analyzed patients undergoing WATCHMAN™ implantation discharged on combined OAC (95% on vitamin K antagonist [VKA]) plus aspirin or antiplatelet therapy (91% on DAPT), using a propensity score-matched design.^61^ At 6 months, rates of non-procedural thromboembolic events and major bleeding were similar in the two groups. While this study suggests that DAPT is not associated with worse outcomes than VKA plus SAPT, this apparent benefit should be interpreted in the context of an outdated comparator, as VKA-based regimens have largely been replaced in contemporary practice by DOAC-based strategies with a more favorable bleeding profile. Additionally, the authors reported that DRT occurred more frequently in patients on antiplatelet therapy (3.1% vs. 1.4%; *P* = .014), a difference that remained significant even after excluding patients discharged on SAPT (3.3% vs. 1.1%; *P* = .005). Whereas current guidelines favor a stepwise strategy, Della Rocca et al. instead explored a sequential, reduced-intensity regimen based on half-dose DOAC (hdDOAC) plus aspirin for 45 days followed by hdDOAC alone. The authors found that, at 13 months, this sequential hdDOAC strategy was associated with significantly lower rates of DRT (0.0% vs. 3.4%; *P* = .009); non-procedural major bleeding (0.5% vs. 3.9%; *P* = .018); and the composite of DRT, thromboembolic events, and major bleeding (1.0% vs. 9.5%; *P* = .002) compared with the standard regimen.^62^ HdDOAC may represent a safer choice in patients undergoing LAAC who have a known contraindication for long-term full-dose anticoagulation. In a large cohort of Asian AF patients, low-dose DOAC was associated with a significantly lower risk of major bleeding compared with VKAs (HR, 0.85; 95% CI, 0.74–0.97).^63^ In the prospective GARFIELD-AF (“Global Anticoagulant Registry in the FIELD-AF”) trial, bleeding rates were lower in underdosed patients compared with those treated with guideline-recommended doses^64^; notably, in low-dosing patients, the rate of major bleeding per 100 person-years was 0.3 (95% CI, 0.2–0.5), confirming the favorable safety profile of reduced-dose DOAC.

Several observations also provide a pathophysiological rationale for this approach. Rodés-Cabau et al.^65^ and Asmarats et al.^66^ showed that LAAC is followed predominantly by activation of the coagulation cascade, with minimal alteration of platelet function: patients treated with OAC had lower coagulation activity and no DRT, whereas DRT events were mainly observed in patients on antiplatelet therapy, who also exhibited higher levels of prothrombotic markers. The ADRIFT (“Assessment of Dual Antiplatelet Therapy versus Rivaroxaban in Atrial Fibrillation Patients Treated with Left Atrial Appendage Closure”) trial randomized 105 patients after successful LAAC to three regimens: 10 mg of rivaroxaban, 15 mg of rivaroxaban, or DAPT. In this study, reduced-dose rivaroxaban was associated with significantly lower thrombin generation compared with DAPT, and no DRT was detected at 3-month follow-up in either rivaroxaban arm, in contrast to the DAPT group (0% vs. 0% vs. 6.1%).^67^ Similarly, the ADALA (“Low-dose Direct Oral Anticoagulation vs. Dual Antiplatelet Therapy After Left Atrial Appendage Occlusion”) study showed that 3 months of low-dose DOAC (2.5 mg of apixaban twice daily) may reduce the composite of major bleeding and thromboembolic events compared with DAPT, without increasing ischemic events.^68^ However, in the recent ANDES trial, short-term DOAC therapy did not significantly lower the incidence of DRT compared with DAPT. Notably, the use of short-term DOAC was associated with a better overall safety profile, mainly driven by fewer bleeding events.^69^ That said, given the lower-than-expected event rates and between-group differences, the study was underpowered for its primary endpoint, and its findings should be interpreted as hypothesis-generating and in need of confirmation by further dedicated trials.

Although these data have not been specifically extracted from populations at the highest risk of bleeding, it is plausible that the benefits of reduced-intensity or reduced-duration DOAC-based strategies may extend to these groups; however, this assumption requires confirmation in dedicated, adequately powered studies. Importantly, it must be noted that even short-term OAC may remain unfeasible in patients with an absolute contraindication to DOAC. In clinical practice, a small but non-negligible proportion of LAAC candidates have such an extreme bleeding risk (eg, diffuse cerebral amyloid angiopathy, prior intracranial hemorrhage, severe hematologic disorders, bowel angiodysplasia, or recurrent gastrointestinal bleeding) that even hdDOAC or DAPT may not be feasible. In major registries, 5%–10% of patients have been discharged on SAPT or no antithrombotic therapy, with overall DRT rates generally low and not consistently different across regimens, although available data are heterogeneous and limited.^70,71^ Notably, in RELEXAO (“Registry on Real-life Experience with Left Atrial Appendage Occlusion”), patients discharged without any antithrombotic treatment had a higher incidence of DRT than those receiving some form of antithrombotic therapy (15.4% vs. 4.5%; *P* = .02), highlighting the potential hazard of complete therapy withdrawal.^72^ Given the observational nature, small sample sizes, and strong selection bias of these studies, SAPT or no antithrombotic therapy after LAAC should therefore be reserved for highly selected patients with absolute contraindications to any OAC- or DAPT-based regimen, after careful Heart Team evaluation of the individual trade-off between thromboembolic and bleeding risk. A final consideration concerns patients with AF and severe renal impairment, in whom the potential benefit of LAAC (namely, the possibility of discontinuing long-term antithrombotic therapy) may outweigh not only the periprocedural risk of the intervention but also those associated with the post-procedural bridging period, until complete device endothelialization has occurred. Patients with CKD are known to have a higher intrinsic bleeding risk due to several mechanisms, including impaired platelet adhesion and activation. A recent analysis from the OCEAN-LAAC (“Optimized Catheter Valvular Intervention – Left Atrial Appendage Closure”) registry supports the feasibility of LAAC in AF patients undergoing hemodialysis (HD).^73^ More specifically, there was no significant difference in the cumulative incidence of major bleeding or ischemic stroke between HD and non-HD patients. However, patients in the non-HD group were older, had a more frequent history of prior bleeding, reported more frequent alcohol use, and had a higher prevalence of hypertension. Therefore, these findings may be biased by baseline differences between groups and should be interpreted with caution. Even though the authors reported that the bleeding event rates did not differ between the two groups during the bridging period, it is not possible to exclude that residual confounding related to the observational, registry-based design may have influenced the reported outcomes and therefore may not fully reflect the true bleeding risk profile in HD patients undergoing LAAC.

Recent data support an overall superior safety profile of rivaroxaban and apixaban compared with VKAs in this population, without an associated increase in stroke risk.^74–76^ Both the AHA^4^ and the Kidney Disease: Improving Global Outcomes (KDIGO) work group^77^ consider apixaban a reasonable alternative to VKA, as it offers a more favorable efficacy-to-toxicity profile. According to the AHA, both full-dose and reduced-dose regimens (5 mg or 2.5 mg twice daily) can be prescribed, whereas KDIGO recommends apixaban only at a reduced dose. Overall, when weighing the benefit of LAAC in advanced CKD, clinicians must consider both the increased thrombotic risk associated with DAPT/DAT during the post-procedural bridging phase and the intrinsically elevated bleeding risk in these patients. An hdDOAC strategy could represent a promising alternative to standard regimens; however, dedicated randomized trials are needed in this specific population to directly compare DAPT, VKA plus SAPT, DOAC, or hdDOAC strategies.

### Device-related thrombus and peri-device leak

DRT and residual PDL represent the most common LAAC device complications in clinical practice. DRT is defined as a homogeneous echo-dense mass adherent to the atrial surface of the LAAC device detected by TEE in multiple projections. DRT incidence appears low, roughly in the 1.5%–4% range varying according to the device type, post-LAAC antithrombotic regimen, and individual registry.^27,38,41,47^ A meta-analysis of 66 studies including 10,154 patients who underwent imaging follow-up after LAAC reported a pooled DRT incidence of 3.8%.^78^ Many factors culminate in DRT, including procoagulant patient-specific factors, choice of antithrombotic agent post-LAAC, and device- and implantation-specific factors. A left ventricular ejection fraction of <40%, female sex, smoking, higher CHA_2_DS_2_-VASc score, spontaneous echocardiographic contrast, pre-existing LAA thrombus, and LAA peak emptying velocity were identified as risk factors.^79–81^ The clinical relevance of the DRT has been challenged by the paucity of available data. The presence of DRT has been linked to an approximately fourfold increase in the risk of ischemic stroke or SE.^21^ In a French registry, DRT occurred in 7.2% of patients and emerged as an independent predictor of ischemic stroke or TIA during follow-up.^74^ It is noteworthy that the impact of DRT on clinical outcomes depends on its characteristics, including size and morphology, as well as the type of antithrombotic therapy after LAAC. However, these features are not always systematically reported, and it is therefore reasonable to assume that not all DRTs carry the same embolic risk. Moreover, despite TEE being considered the gold-standard methodology for DRT diagnosis, CCTA is emerging as a valuable complementary imaging tool, as its superior spatial resolution allows a more accurate reconstruction of DRT morphology and may help differentiate thrombi with higher versus lower embolic potential^82^; this could, in the future, allow a more refined characterization of DRT and is likely to inform risk stratification and, consequently, the management of these patients. In addition, antithrombotic therapy is usually initiated or intensified at the time DRT is detected, making it difficult to clarify whether the subsequent low rate of ischemic events is driven by the natural history of DRT or by the therapeutic intervention. The most widely used management approach consists of initiating warfarin for 8–12 weeks, targeting an international normalized ratio (INR) of 2.0–3.0 in patients who were not on OAC at the time DRT was detected. In those already treated with warfarin, increasing the INR target to 2.5–3.5 is advised, and low-dose aspirin can be added in patients with an acceptable bleeding risk.

As an alternative, a full-dose DOAC regimen for 8–12 weeks, with or without concomitant low-dose aspirin, may be employed; in this setting, most of the available experience relates to apixaban and rivaroxaban, whereas dabigatran has been suggested to be less effective in the setting of thrombus formation on foreign material. In cases of particularly large (>15 mm) and mobile thrombi, the perceived embolic risk may justify the use of low-molecular-weight heparin and earlier repeat imaging (eg, at 2 weeks) to promptly assess treatment efficacy.^83^ If DRT persists despite optimized antithrombotic therapy and there is concern about high embolic risk or recurrent ischemic events, surgical extraction of the device and associated thrombus may be considered in carefully selected patients. More recently, at our institution, percutaneous removal of DRT from WATCHMAN™ devices using dedicated thrombaspiration systems has been achieved, thus offering a possible interventional alternative in this complex clinical scenario. However, the efficacy and safety of this strategy need to be confirmed in larger, prospective cohorts, and our experience should therefore be regarded as preliminary, exploratory proof of concept.

PDL is defined as any residual communication between the LAA and the left atrium following LAAC. The reported incidence of PDL varies widely across studies, ranging from 10% to 54%.^27,47^

Several factors may explain these discrepancies, including the dimensional criteria used to define PDL (<3, ≥3, <5, ≥5 mm), the type of follow-up imaging modality, and the timing of surveillance.^31,84^ Notably, the size thresholds adopted to define and classify PDLs often differ according to the imaging system employed. In particular, several studies suggest that cardiac computed tomography may outperform TEE in PDL detection and that a temporal trend exists, with a tendency to shrink over time.^31,85^ This may account for some of the differences reported, as there is still no consensus on the preferred imaging modality or the optimal timing for follow-up. Most centers tend to favor follow-up imaging scheduled before any change in antithrombotic therapy, to decide whether to proceed with de-escalation or to continue the current regimen. Additionally, it must be noted that the technological evolution of LAAC devices and implantation techniques is likely to have influenced contemporary PDL rates.^27,47^ To date, several limitations arise when considering the residual thromboembolic risk of patients who have undergone LAAC and harbor a PDL. First, most studies do not report the underlying mechanism of the PDL (eg, peri-device gap, incomplete ostial coverage, fabric permeability) but rather evaluate it solely on the basis of its dimension. Accordingly, there is still debate about what PDL size should be considered clinically relevant, and emerging data suggest that any residual leak, regardless of its size, may not be completely benign.

The currently used threshold stems from the definition of effective LAA exclusion adopted in the PROTECT AF and PREVAIL trials, in which LAAC was considered successful when the residual leak was <3 mm (±2 mm). Moreover, at the time of PDL detection, clinicians frequently adjust the duration and/or intensity of antithrombotic therapy, which reduces the subsequent incidence of stroke or SE. Patients with large leaks often remain on long-term anticoagulation or undergo PDL closure, and therefore the assessment of the “natural” impact of residual leak in this subgroup is heavily confounded by treatment bias. Because of these limitations, most published studies on the clinical impact of PDL have been underpowered and/or inconclusive. Three complementary studies have shown that even small PDLs after LAAC are not clinically benign. In a large US registry, small residual leaks of <5 mm were present in about one-quarter of patients and were associated with modestly increased risks of thromboembolic events, major bleeding, and major adverse events.^21^ In the pooled 5-year PROTECT AF/PREVAIL cohorts, small leaks persisting at 1 year nearly doubled the rate of stroke or systemic embolization, mainly due to an excess of non-disabling stroke.^19^ Consistently, in the Amulet™ IDE trial, PDL ≥3 mm at 45 days independently correlated with a higher incidence of ischemic stroke, systemic embolization, or CVD.^86^ Collectively, these data support a graded, unfavorable prognostic impact of even small PDLs after LAAC. The management of PDL depends on center expertise, the likelihood of temporal regression (which it is itself related to leak size), and the tolerability of OAC.

In patients with an absolute contraindication to OAC, who represent the main target population for LAAC in Europe, initiating or prolonging anticoagulation may not be a viable option. In such cases, a more cautious watchful-waiting strategy, with repeat imaging surveillance, may be advisable, particularly in the presence of small PDLs.^87^ Interventional options to close larger PDLs include the use of vascular plugs and cardiac occluders, detachable coils, radiofrequency ablation, or a combination of these techniques.^88–90^ The choice of strategy is primarily driven by local experience, and no single approach has clearly emerged as superior. Nonetheless, certain PDL anatomies may favor specific techniques: narrower and shallower leaks are often more amenable to coil embolization, whereas broader neck defects or unprotected accessory lobes are typically treated with plugs or a second occlusion device.^87^ Across published series, the acute procedural success of PDL closure has been consistently high, with low rates of major complications regardless of the technique employed. Despite a significant reduction in leak size in most patients, persistent flow into the LAA on follow-up imaging has been reported in 13%–39% of cases.^91^

Important uncertainties remain regarding the long-term management of patients undergoing interventional PDL closure. There are no standardized protocols for post-procedural imaging surveillance or for antithrombotic therapy after PDL closure. In most studies, patients were treated with antiplatelet agents and underwent only a single follow-up imaging assessment. Although the low incidence of ischemic events reported in these cohorts supports the potential effectiveness of PDL closure, the small sample size and limited follow-up duration preclude firm conclusions. Furthermore, the impact of placing multiple devices within the LAA on the risk of DRT remains unclear and warrants dedicated investigation.

## Emerging left atrial appendage closure technologies

While current percutaneous LAAC devices such as WATCHMAN FLX™/FLX™ Pro and Amplatzer™ Amulet™ dominate clinical practice, several next-generation technologies are in development or early clinical use. These systems have been designed with the aim of improving anatomical adaptability, procedural safety, and long-term outcomes, particularly in complex anatomies or in patients at very high bleeding risk. Most of these devices remain investigational, with US Food and Drug Administration (FDA) approval under evaluation, and available human data are limited in size and follow-up. Their ultimate role will depend on procedural feasibility (including the learning curve), imaging requirements and follow-up, and the definition of safe and effective post-procedural antithrombotic regimens.

### Append implant-free left atrial appendage closure system (Append Medical)

Append Medical (Or Yehuda, Israel) is a medical device company developing an investigational implant-free solution to close the LAA. Following transseptal access into the left atrium, the tissue grabber is advanced into the LAA and captures the LAA tissue using vacuum suction. The grabber then pulls and invaginates the LAA into a ligation device positioned at the LAA ostium. This device releases and tightens the suture around the invaginated LAA tissue, with the aim of achieving a mechanical seal. The sealing mechanism is intended to ensure tissue-to-tissue apposition, promoting natural healing and potentially permanent closure over time. Once the suture is secured, a suture cutter is inserted over the suture to trim the excess material above the knot. The entire system is then retrieved, leaving no implant or foreign material in the body. This approach may offer potential advantages, including the possibility of LAA elimination with no device left in the heart and a theoretically broad applicability to a wide range of different anatomies. The procedure can be performed as a standalone treatment or in conjunction with AF ablation, and it can be guided using intracardiac echocardiography imaging, potentially providing flexibility and adaptability across different clinical scenarios. Currently, the Append closure device is not yet FDA-approved, and no human clinical studies have been publicly reported. Consequently, its procedural learning curve, optimal imaging and follow-up strategy, and post-procedural antithrombotic regimen, as well as its long-term safety and efficacy profile (including PDL, DRT, and bleeding risk), remain to be defined.

### Conformal Left Atrial Appendage Seal (Conformal Medical)

The Conformal Left Atrial Appendage Seal (CLAAS) from Conformal Medical (Nashua, NH, USA) represents a novel concept in LAAC, a departure from traditional plug or lobe-disc designs.^92^ It features a self-expanding, spherical nitinol endoskeleton enveloped by a porous polyurethane–carbonate foam cup, designed to conform to the LAA ostium and accommodate a wide range of anatomies, including shallow or irregular LAA morphologies. The device anchors at the ostial level rather than within the distal LAA, potentially simplifying implantation and reducing complications such as perforation or embolization. A 360° PET-covered surface is designed to enhance endothelialization and minimize PDLs, while a PTFE membrane on the proximal surface provides a low thrombogenic atrial interface. The implant is available in two sizes (27 and 35 mm), is fully recapturable prior to release, and includes two rows of anchor barbs at the mid-portion of the frame to ensure stable fixation. Preclinical and initial human results suggest feasible implantation with encouraging sealing and device stability, with low observed rates of DRT in the available, relatively small cohorts.^92,93^ However, these data are limited in sample size and follow-up duration, and the CONFORM Pivotal trial (“An Evaluation of the Safety and Effectiveness of the Conformal CLAAS System for Left Atrial Appendage Occlusion”) (NCT05147792) will be critical to define its comparative efficacy; safety; and long-term outcomes, including PDL, DRT, and bleeding events, versus WATCHMAN™ and Amulet™ systems.

### Laminar (J&J MedTech)

The Laminar occluder from J&J MedTech (New Brunswick, NJ, USA) is a recently developed device that, unlike traditional plug-based systems, eliminates the LAA using a rotational mechanical action rather than simply sealing it. The Laminar implant features two elements: a self-expanding nitinol ball and a lock. The atraumatic ball is available in two sizes (12- and 16-mm diameter) and, when deployed, expands to a sphere with a flat distal portion that can be advanced safely into the LAA without any side hooks or barbs. The lock is flower-shaped with six “petals” aligned and keyed to interlock with the ball to achieve tissue compression and seal. The atrial-facing lock is 16 mm in diameter for both devices. This design is intended to isolate the LAA, while minimizing the surface area exposed to blood flow and, therefore, may help to reduce the risk of thrombus formation and PDLs. A key feature of the device is its low-profile flow-diverting mesh design, acting more as an endothelialization scaffold than as a plug. This theoretically promotes biologic integration and potentially reduces both inflammation and thrombogenicity. In the first-in-human feasibility study, the Laminar device was implanted, achieving 100% procedural success. At 12-month follow-up, no DRTs, residual PDLs, or major adverse events were reported.^94^ These early results in a limited number of patients are encouraging but should be interpreted with caution given the small sample size and short follow-up. The Laminar device is now undergoing a large-scale pivotal trial (LAAX [“Laminar Left Atrial Appendage Elimination”] IDE study), which aims to compare Laminar to currently FDA-approved LAA occluders, with long-term follow-up extending through 2032.

### LAmbre™ (LifeTech Scientific Corp.)

The LAmbre™ device from LifeTech Scientific Corp. (Shenzhen, China) is a self-expanding device made of a nitinol mesh and covered PET membranes, offering a double sealing and stabilization mechanism. Structurally, LAmbre™ consists of two main components: a distal lobe (umbrella) and a proximal disc (cover). The distal lobe features two rows of eight perimetric anchors and eight small distal hooks that embed into the LAA wall, supported by a mild radial force for enhanced fixation.^95^ The proximal disc covers the LAA ostium and incorporates a recessed proximal screw to minimize the risk of DRT. A short, articulating central waist connects the lobe and the disc, allowing them to align independently and conform to complex anatomies without compromising device stability. LAmbre™ is available in two configurations to accommodate both single-lobe and double-lobe LAA anatomies. With a total device length of <10 mm, LAmbre™ may be particularly useful for patients with shallow LAA morphologies.^96^

### Occlutech left appendage closure device (Occlutech)

The Occlutech device (Occlutech Holding AG, Schaffhausen, Switzerland) consists of a self-expanding, flexible nitinol cylindrical-shaped mesh that adapts to the shape of the LAA. The proximal diameter of the device is larger than the distal one, and this contributes to obstructing the neck of the LAA and sealing the ostium. The loops at the distal rim aim to keep the implanted device in a stable position and are less traumatic than barbs, with the aim of reducing the risk of perforation and pericardial effusion.^97^ The outer surface of the occluder is covered with a polyurethane membrane that allows exclusion of blood flow. As an insufficient anchoring of the device was identified as a safety concern during the first clinical trial, in the second generation of the device, additional anchors were added to the design that support distal loops to ensure safe fixation in the LAA.^98^

### Ultraseal (Cardia)

The Ultraseal LAAC device (Cardia, Inc., Minnetonka, MN, USA) is a fully retrievable self-expandable nitinol device composed of two parts: a soft distal bulb that anchors the device to the LAA through 12 stabilizing hooks and a three-leaflet multilayered sail with a proximal polyvinyl alcohol foam and a distal polyester layer for LAA occlusion. These elements are connected by a dual articulating joint, enabling multidirectional movement and optimal adjustment to different ostium angles and shapes.^66^ Clinical experience with Ultraseal remains relatively limited, and additional studies are needed to characterize long-term safety; durability of occlusion; and the balance between stroke prevention, DRT, and bleeding under different antithrombotic strategies.

## Conclusion

LAAC has undergone a remarkable evolution from a marginal surgical intervention to a clinically validated guideline-endorsed option for stroke prevention in patients with NVAF. Today, LAAC stands as a valuable alternative or adjunct to anticoagulation therapy, particularly for patients with a high bleeding risk or absolute contraindications to long-term oral anticoagulants. This transformation has been driven by technological innovation. New-generation devices now offer enhanced safety, improved anatomical conformability, and more reliable occlusion, thanks to features such as advanced anchoring systems, biocompatible surfaces that promote rapid endothelialization, and strategies that eliminate intracardiac implants altogether. These advancements have reduced procedural complications and minimized residual leaks and DRT, while expanding applicability to a broader range of anatomies and clinical scenarios.

Trials such as PRAGUE-17 and OPTION suggest that LAAC can provide stroke prevention comparable to DOACs while reducing bleeding in carefully selected patients, including those undergoing catheter ablation, thereby supporting its role as a valuable alternative or adjunct to long-term anticoagulation.

At the same time, recent evidence shows that the net clinical benefit of LAAC is not uniform across all high-risk patients. In very elderly individuals with extreme baseline thromboembolic and bleeding risk, the combination of procedural complications and the burden of post-procedural antithrombotic therapy may offset the expected benefit in stroke reduction. These findings support the notion that outcomes after LAAC are critically dependent on three tightly interrelated factors: appropriate patient selection, procedural safety, and optimization of post-implant antithrombotic regimens. In this context, LAAC should be viewed less as a one-size-fits-all alternative to anticoagulation and more as a component of a personalized, risk-adapted strategy.

Looking ahead, ongoing randomized trials across the risk spectrum are expected to refine the indications, timing, and therapeutic combinations for LAAC. In parallel, emerging implant-based and implant-free technologies aim to further enhance procedural efficacy, expand anatomical eligibility, and minimize long-term bleeding by enabling shorter and less intensive antithrombotic regimens. As these data mature, LAAC may consolidate its role from a niche intervention reserved for “no-option” patients to an integral element of comprehensive AF management, provided its use remains grounded in careful, individualized assessment of the trade-offs between stroke prevention, bleeding risk, and procedural safety.
